# Prevalence of mental distress and associated factors among Hawassa University medical students, Southern Ethiopia: a cross-sectional study

**DOI:** 10.1186/s13104-016-2289-7

**Published:** 2016-11-08

**Authors:** Biniam Melese, Birhanu Bayu, Fikir Wondwossen, Kalkidan Tilahun, Seti Lema, Moges Ayehu, Eskindir Loha

**Affiliations:** 1 School of Medicine, Hawassa University, Hawassa, Ethiopia; 2 School of Public and Environmental Health, Hawassa University, Hawassa, Ethiopia

**Keywords:** Mental distress, Medical students, College students, Common mental disorders

## Abstract

**Background:**

Mental distress is a mental health problem expressed with variable levels of depressive, anxiety, panic or somatic symptoms. Owing to several factors tertiary level students are among the population with higher prevalence of mental distress and an even more higher prevalence has been reported in medical students. The aim of this study was to determine the prevalence of mental distress among medical students, and to evaluate contextually relevant associated factors.

**Methods:**

A cross-sectional study was conducted among medical students attending Hawassa University College of Medicine and Health Sciences in 2013/2014 academic year. Stratified random sampling was implemented with each strata representing the year of study of the students. Data on mental distress was collected using the Self-Reporting Questionnaire-20 (SRQ-20). Data was entered into and analyzed using IBM SPSS statistics 21. A cut-off point of 8 and above was used to classify students as having mental distress.

**Results:**

Among 240 students included in the study, 72 (30%) of them were found to have mental distress. There was no significant difference in mental distress between males and females (COR = 1.18, 95% CI = 0.62–2.25). On bivariate analysis, students with age less than or equal to 21 years showed higher odds of having mental distress (COR = 2.3, 95% CI: 1.26–4.22), but because of having high correlation with students’ year of study, age was excluded from the multivariate model. In this study being a pre-medicine student (AOR = 3.61, 95% CI: 1.45–8.97), perceiving medical school as very stressful (AOR = 3.89, 95% CI: 1.52–9.94), perceiving living environment as very crowded (AOR = 2.43, 95% CI: 1.24–4.77) and having a feeling of insecurity about one’s safety (AOR = 2.93, 95% CI: 1.51–5.68) had statistically significant association with mental distress.

**Conclusions:**

In this study one-third of medical students were found to have mental distress. Designing prevention and treatment programs to address contextually relevant factors is very important.

## Background

Individuals with mental distress present with different levels of depression, anxiety or somatic symptoms. These symptoms significantly interfere with their relationships with other people, their work, and enjoyment of life. Having mental distress can be difficult for the person, family and colleagues [1, 2]. Depression in young adults is a serious public health problem, and at the same times a source of immense human suffering [3]. Many studies also reported the high prevalence of mental distress among college students compared to the general population [4]; in particular it is more common among medical students owing to the unique setting of their living condition which exposes them to other dynamics such as added stress with academic challenge, social interaction within the mixed cultural pool and separation from pre-existing social support [5–7]. Lislotte et al. [8] extensively summarized 40 papers published from 1980 to 2005 that were conducted among US and Canadian medical students and concluded that mental distress was highly prevalent among medical students compared to the general population and age-matched peers. In this study mental distress was found more common in female medical students than male medical students. With regard to student populations, it is recognized as a common and debilitating disturbance, whilst related symptoms are evident in all areas of functioning, including motivation, concentration, perception of self-worth and mood [9]. The Eisenberg study, a large cross-sectional study from the United States, indicated that 15.6% of undergraduates and 13.0% of graduate students reported suffering a depressive or anxiety disorder. Depression alone was reported by 13.8% of undergraduates and 11.3% of graduate students, with no major difference between males and females. Anxiety disorders were less common, being prevalent in 4.2% of undergraduates and 3.8% of graduate students [10]. Studies conducted in sub-Saharan Africa show that 20–30% of primary health clinic attendees present with depressive symptoms and other psychological disorders as the first or secondary reason for seeking medical care [11–13]. There are limited studies carried in Ethiopia to estimate the prevalence of mental distress in the general population using Self-Reporting Questionnaire (SRQ-20). These studies showed the prevalence of mental distress ranges from 11.7 to 17.7% [14, 15]. Moreover, there are also few studies conducted among college students to see the prevalence of mental distress [16–19]. The one month prevalence of mental distress reported in these studies was ranged between 21.6 and 49.1%. However, except one study conducted among Addis Ababa University medical students, the remaining three studies included undergraduate university students attending different departments. Furthermore, the previous mentioned study among Addis Ababa University medical students was also conducted in 2001, which may not exactly reflect the current Ethiopian medical students’ situation. In the aforementioned studies prevalence of mental distress was found higher in those students with female sex, a family history of mental illness, frequent conflicts with their peers, never attending religious programs, substance users, being preclinical students, being young age, lack of adequate access to sanitary and recreational service, living in a very crowded environment and being insecure about their safety. Not having adequate income, difficulty in making friends and dating were also found associated with mental distress. In light of the fact that there were few literatures concerning the issue in our country, particularly among medical students, this study was conducted with an aim of providing baseline information for further study and to offer an input for the responsible bodies to plan an intervention for this group of population.

### Objectives

The primary objective of the study was to assess the prevalence of mental distress among medical students, and to evaluate contextually relevant associated factors.

## Methods

### Study design and setting

A facility based cross-sectional study was conducted at Hawassa University College of Medicine and Health Sciences, which is 270 km south of Addis Ababa. The college is one of the 7th colleges under Hawassa University. The college is established to teach medical, nursing, public and laboratory students. In the academic year 2013/2014 the college had enrolled a total of 2408 students. From these students 1289 of them were medical students. Medical students are expected to stay in the college for 6 years. There are four major phases medical students have to pass during their 6 years study periods: pre-medical (6 months), preclinical (nearly 2 years), clinical (two and half years) and internship (1 year). During pre-medical phase students are expected to take courses like Medical Ethics and Legal Medicine, Medical Psychology, Information Communication Technology, Medical Anthropology and Sociology, Sophomore English and Civics and Ethical Education, and Environmental Health. During their preclinical phase major basic sciences courses like Anatomy, Physiology, Biochemistry, Pharmacology, Pathology and Microbiology together with other relevant courses will be covered. During clinical years students will have both theoretical and practical session exposure for clinical major courses like Internal Medicine, Surgery, Gynecology/Obstetrics, Pediatric and Child Health together with minor clinical courses like Psychiatry, Dermatology, Ear Nose Throat (ENT), Ophthalmology, Radiology, and Dentistry. The Medical Schools use letter and grade point scale based on Hawassa University Senate Legislation [20]. In order to be promoted to the next level and graduate successfully with Medical Doctorate Degree, a student should obtain at least 2.00 points Cumulative Grade point Average (CGPA) out of maximum score of 4.00 points.

### Participants

The participants of this study were undergraduate medical students enrolled in the year 2013/2014 and attending classes in regular program under School of Medicine. Students were stratified based on the year of study. Sample size was calculated by anticipating the prevalence of mental distress as 21.6% (based on recent study conducted in Adama University [13]), 95% confidence level and 5% margin of error. Considering the total population (N = 1289 medical students), correction factor was used and adding 10% for non-response made our final sample size 240. To account for the relative size of students in different years of studies, proportional allocation technique was employed. Based on this, majority of students were from pre-medicine year (n = 68 students) while the least were taken from clinical year II (n = 20). The students from each class year were randomly selected. Students who formally enrolled but not attending class during study periods were excluding from the study.

### Assessment

Data was collected from May 12–26/2014. The periods were exam free periods. Assessments consist of an evaluation of basic socio-demographic characteristics, educational background, history of substance use, respondents’ monthly income, students’ attitude towards medical school, satisfaction by school sanitation and recreational center, students’ Cumulative Grade point Average (CGPA), relationship with their fellows, attitude about living room and their safety. Variables were compiled based on previous studies conducted in Ethiopia. Presence of mental distress was evaluated using a 20 items self-administered structured Self-Reporting Questionnaire (SRQ-20). Self-Reporting Questionnaire (SRQ-20) was originally developed by World Health Organization (WHO) [21] to detect presence of mental distress and validated in developing country including Ethiopia [22–24]; and found to be highly sensitive and specific. In this study a cut-off point 8 and above was taken as having mental distress based on previous finding [23]. Since medium instruction in Ethiopian higher education is English we used the English version of the instruments.

### Data management

Data were collected by final year medical students after thoroughly discussed about data collection procedures. Data was collected at the end of each class. Moreover, study participants were also informed to fill the questionnaire independently so that bias may be minimized. Data was entered into and analyzed using IBM SPSS statistics 21. Variables with a *P* value less than 0.05 were declared to have a statistically significant association. Each independent variable against the dependent variable was tested for having statistical significant association using binary logistic regression. Multivariable logistic regression analysis was done for those variables with P-value less than or equal to 0.25 during binary logistic regression analysis. Age was highly correlated with year of study (r = 0.8) and removed from the multivariate model.

## Result

### Socio-demographic characteristics of the participants

A total of 240 medical students were assessed, of which 178 (74.2%) were males. 68 (28.3%) of them were pre-medicine students while 91 (37.9%) and 81 (33.8%) of them were pre-clinical and clinical students respectively. Respondents’ age ranged from 18 to 29 years, with a mean (SD) of 21.3 (2.11) years. Majority of the participants (58.8%) were followers of Orthodox religion, followed by protestant (27.5%), Muslim (9.6%), and others (4%). 157 (65.4%) of the respondents’ Cumulative Grade Point Average (CGPA) were above 3.00. Students’ monthly income ranged 50–3000 Ethiopian Birr. During analysis their income was grouped into tertiles and 100 (41.7%) of students had income less than 400 Ethiopian Birr (Table 1). Except intern (last year medical students), the income source of majority (85.4%) students was immediate family members.

**Table 1:** Distribution of students by socio-demographic and economic characteristics

Characteristics	Number	(%)
Sex		
Male	178	74.0
Female	62	25.5
Age		
≤21	145	60.4
>21	95	39.6
Year of study		
Pre-medicine	68	28.3
Pre-clinical	91	37.9
Clinical	81	33.8
Religion		
Orthodox	141	58.8
Protestant	66	27.5
Muslim	23	9.6
Others	10	4.0
Monthly income in Birr		
≤400	100	41.7
401–700	66	27.5
≥701	74	30.8
Cumulative grade point average (CGPA)		
>3.50	70	29.2
3.00–3.50	87	36.2
2.50–3.00	67	27.9
2.00–2.50	15	6.2
<2.00	1	0.4

Birr: 1 USD = 20 Birr

### Social interaction, attitude towards the college environment and prevalence of substance use

Two hundred twenty-eight (95%) rated their relationship with their fellows either very good or good. Meanwhile, 192 (80%) of the students reported they were not in romantic relationship during study periods. 209 (87%) medical students had a habit of visiting religious place at least once in two weeks’. In addition, 174 (72.5%) students felt medical school is stressful than other disciplines. Majority of students were dissatisfied by sanitation and recreational center under the college [156 (65%) and 196 (81.7%) of them respectively]. More than half, 128 (53.3%) of students perceived their living condition are overcrowded while 77 (32.1%) had feeling of insecurity about their safety. Among study participants, 41 (17.1%) used one of the three substances commonly used in Ethiopia (khat, cigarette, or alcohol).

### Mental distress and associated factors

Prevalence of mental distress using SRQ-20, with a cut-off point of 8 and above was 30% (72 students out of 240 students). The mean value for the total score was 6.1 with a standard deviation of 4. The total scores were in the range of 0–17. There was no significant difference of mental distress between males and females (COR = 1.18, 95% CI: 0.62–2.25). On bivariate analysis students with age less than or equal to 21 showed higher odds of having mental distress (COR = 2.3, 95% CI: 1.26–4.22). Since age had high correlation with students’ year of study, age was excluded from the multivariate model. Being a pre-medicine student (AOR = 3.61, 95% CI: 1.45–8.97), perceiving medical school as very stressful (AOR = 3.89, 95% CI: 1.52–9.94), experiencing living environment as very crowded (AOR = 2.43, 95% CI: 1.24–4.77), feeling of insecurity about their safety (AOR = 2.93, 95% CI: 1.51–5.68) were found statistically significant association with mental distress. Even if, on bivariate analyses, dissatisfaction with university sanitation (COR = 2.65, 95% CI: 1.39–5.06) and recreational facility (COR = 2.62, 95% CI: 1.11–6.20) showed higher odds of having mental distress, on multivariate analysis no statistically significant associations were found (Table 2).

**Table 2:** Bivariate and multivariate analyses of factors associated with mental distress

Variables (n = 240)	Total symptoms score^a^		OR (95% CI)	
	≥8	<8	Crude	Adjusted
	No. (%)	No. (%)		
Sex				
Male	55 (76.4)	123 (73.2)	1.18 (0.62–2.25)	NA
Female	17 (23.6)	45 (26.8)	1	
Age group^b^				
≤21	53 (73.6)	92 (54.8)	2.30 (1.26–4.22)**	NA
>21	19 (26.4)	76 (45.2)	1	
Students monthly income in Birr				
≤400	36 (50.0)	64 (38.1)	1	1
401–700	19 (26.4)	47 (28.0)	0.72 (0.37–1.41)	0.58 (0.26–1.27)
≥701	17 (23.6)	57 (33.9)	0.53 (0.27–1.05)	0.52 (0.22–1.26)
Feeling towards their GPA				
Happy and satisfied	27 (37.5)	67 (39.9)	1	NA
Happy but I can do better	32 (44.4)	83 (49.4)	0.96 (0.52–1.75)	
Not happy	13 (18.1)	18 (10.7)	1.79 (0.77–4.16)	
Visiting religious place				
At least once in two weeks	62 (86.1)	147 (87.5)	1	NA
Occasionally	6 (8.3)	14 (8.3)	0.74 (0.21–2.61)	
Never	4 (5.6)	7 (4.2)	0.75 (0.16–3.56)	
Year of study				
Pre-medicine	30 (41.7)	38 (22.6)	3.78 (1.79–7.99)**	3.61 (1.45–8.97)**
Pre-clinical	28 (38.9)	63 (37.5)	2.13 (1.03–4.41)*	1.55 (0.64–3.72)
Clinical	14 (19.4)	67 (39.9)	1	1
Relationship with fellows				
Very good	19 (26.4)	60 (35.7)	1	NA
Good	49 (68.1)	100 (59.5)	1.55 (0.83–2.87)	
Poor	4 (5.6)	8 (4.8)	1.58 (0.42–5.83)	
Perception of medical school				
Very stressful	26 (36.1)	28 (16.7)	3.16 (1.44–6.92)**	3.89 (1.52–9.94)**
Stressful	31 (43.1)	89 (53.0)	1.18 (0.58–2.40)	1.20 (0.54–2.68)
Not stressful at all	15 (20.8)	51 (30.4)	1	1
Attitude towards sanitary facility				
Dissatisfied	57 (79.2)	99 (58.9)	2.65 (1.39–5.06)*	1.95 (0.95–4.02)
Satisfied	15 (20.8)	84 (35.0)	1	1
Attitude to recreational facility				
Dissatisfied	65 (90.3)	131 (78.0)	2.62 (1.11–6.20)*	1.78 (0.67–4.75)
Satisfied	7 (9.7)	37 (22.0)	1	1
Feeling towards living room				
Overcrowded	51 (70.8)	77 (45.8)	2.87 (1.59–5.19)***	2.43 (1.24–4.79)**
Not overcrowded	21 (29.2)	91 (54.2)	1	
Feeling towards their safety				
Feel insecure	39 (54.2)	38 (22.6)	4.04 (2.25–7.28)***	2.93 (1.51–5.68)***
Feel secure	33 (45.8)	130 (77.4)	1	1
Substance use				
At least one substance	12 (16.7)	29 (17.3)	0.96 (0.46–2.01)	NA
Not at all	60 (83.3)	139 (2.7)	1	

Birr: 1 USD = 20 Birr

*NA* Not applicable (excluded from the multivariate model since their P value was greater than 0.25)

* P < 0.05, ** P < 0.01, and *** P < 0.001

^a^≥8 was categorized as mentally distressed

^b^Age was highly correlated with year of study (r = 0.8) and removed from the multivariate model

## Discussion

One-third of the study participants were found to be mentally distressed. This study confirmed the high prevalence of mental distress among medical students than the general population which was reported by Kebede et al. [14] and Gelaye et al. [15] as 11.7 and 17.7% respectively. A number of factors including academic pressure, excessive work load, sleep deprivation exposure to patients’ suffering and deaths and lack of adequate holiday break [25, 26] could be reason why mental distress is very common in medical students than the general population. Though the periods and the setups are different from previous studies, the finding of this study is consistent with similar kind of study done on medical students at Addis Ababa University by Alem et al. (32.6%) [16]. On the other hand, mental distress prevalence in our study is lower than the previous studies done in Gondar and Hawassa University [19, 27] and higher than the study done at Adama University [18] (40.9, 49.1 and 21.6%, respectively). These differences could be attributed to the socio-cultural and environmental factors, or the difference in the SRQ-20 cut-off points we used and year of the study.

Unlike the previous studies [8, 27] students’ gender was not found associated with mental distress in our study. Even if no statistical significant association; in this study male were more likely to report mental distress than the previous studies which reported mental distress more in female students. Our study shows the risk of developing mental distress decreases as year of study advances in the medical school. This finding is consistent with previous study done on medical students at Addis Ababa University by Alem et al. [16]. In this study the odds of developing mental distress in pre-medicine students is 3.61 times higher than students in clinical years. Adjustment difficulty to the new environment, new academic challenges, being separated with their close family and old friends might explain why mental distress is common in early years. Moreover, adjustment difficulty to the academic challenge, new social interaction within the mixed cultural pool and separation from pre-existing social support could have their own contribution for making mental distress high among medical students.

Like the previous studies [18, 19] perceiving medical school as stressful, perceiving the living environment as crowded, feeling of insecurity about their safety are associated with students’ mental distress. The odds of developing mental distress with perceiving medical school as very stressful, feeling living environment as crowded, feeling of insecurity about their safety was 3,89, 2.43, and 2.93 respectively. On the other hand history of substance use, students’ monthly income, not visiting religious area, relationship with their fellows, dissatisfaction by university sanitary and recreational facility were not found as a risk factors unlike previous studies [16, 18, 19].

The findings of this study should be taken with caution considering the lack of data on those students who did miss their class–for unknown reasons. If these students were systematically different from those who were in the class at the time of data collection, it might have affected the study findings. Moreover, the study was conducted among medical students of only one institution; the result may not be generalizable to all students attending higher Ethiopian institutions. Nevertheless, the result is indicative of the current status of mental distress among medical students attending institutions of similar setups.

## Conclusions

One-third of medical students reported having mental distress; confirming the presence of mental distress in medical students being high. The likelihoods of having mental distress were higher among students who perceive medical school as stressful, living environment as crowded, and feeling insecure about their safety. Designing prevention and treatment programs to address contextually relevant factors is very important.

## Abbreviations

AOR: adjusted odds ratio
COR: crude odds ratio
CGPA: Cumulative Grade Point Average
SRQ: self-reporting questionnaire
